# RETRACTION: Role and Mechanism of Circ‐PDE7B in the Formation of Keloid

**DOI:** 10.1111/iwj.70743

**Published:** 2025-08-06

## Abstract

**RETRACTION:** TangY. and LiX. , “Role and Mechanism of Circ‐PDE7B in the Formation of Keloid,” International Wound Journal 20, no. 9 (2023): 3738–3749, 10.1111/iwj.14269.37291755 PMC10588313

The above article, published online on 08 June 2023 in Wiley Online Library (http://onlinelibrary.wiley.com/), and its corrigendum (https://doi.org/10.1111/iwj.14869), has been retracted by agreement between the journal Editor in Chief, Professor Keith Harding; and John Wiley & Sons, Ltd. The original published article did not include details about the location for sample collection and details about the organization which provided ethical approval for the study. In addition, individuals other than the authors were included in the author contributions statement, which casts doubt about the origins of the research and authorship for this article. A corrigendum was published on 25 April 2024 (https://doi.org/10.1111/iwj.14869) which stated that samples were collected at Xi'an Central Hospital and corrected the authorship contribution statement to include reference to the listed authors. However, no information regarding the ethical approval of the study was included.

The authors did not respond to an inquiry by the publisher and a request for original data and ethics approval information. As such, the publisher is not able to verify the origins and veracity of the research presented in this article. The editors have therefore agreed to issue a retraction for the article. The authors did not respond to our notice regarding the retraction.

**RETRACTION:** Y. Tang and X. Li , “Role and Mechanism of Circ‐PDE7B in the Formation of Keloid,” International Wound Journal 20, no. 9 (2023): 3738–3749, 10.1111/iwj.14269.37291755 PMC10588313

The above article, published online on 08 June 2023 in Wiley Online Library (http://onlinelibrary.wiley.com/), and its corrigendum (https://doi.org/10.1111/iwj.14869), has been retracted by agreement between the journal Editor in Chief, Professor Keith Harding; and John Wiley & Sons, Ltd. The original published article did not include details about the location for sample collection and details about the organization which provided ethical approval for the study. In addition, individuals other than the authors were included in the author contributions statement, which casts doubt about the origins of the research and authorship for this article. A corrigendum was published on 25 April 2024 (https://doi.org/10.1111/iwj.14869) which stated that samples were collected at Xi'an Central Hospital and corrected the authorship contribution statement to include reference to the listed authors. However, no information regarding the ethical approval of the study was included.

The authors did not respond to an inquiry by the publisher and a request for original data and ethics approval information. As such, the publisher is not able to verify the origins and veracity of the research presented in this article. The editors have therefore agreed to issue a retraction for the article. The authors did not respond to our notice regarding the retraction.

